# Age-Related Changes in Malaria Clinical Phenotypes During Infancy Are Modified by Sickle Cell Trait

**DOI:** 10.1093/cid/ciab245

**Published:** 2021-03-19

**Authors:** Nicholas Zehner, Harriet Adrama, Abel Kakuru, Teddy Andra, Richard Kajubi, Melissa Conrad, Felistas Nankya, Tamara D Clark, Moses Kamya, Isabel Rodriguez-Barraquer, Grant Dorsey, Prasanna Jagannathan

**Affiliations:** 1 Department of Medicine, Stanford University, Stanford, California, USA; 2 Infectious Diseases Research Collaboration, Kampala, Uganda; 3 Department of Medicine, University of California, San Francisco, California, USA; 4 Department of Medicine, Makerere University College of Health Sciences, Kampala, Uganda; 5 Department of Microbiology and Immunology, Stanford University, Stanford, California, USA

**Keywords:** malaria in infancy, asymptomatic parasitemia, sickle cell trait

## Abstract

**Background:**

Infants are protected against *Plasmodium falciparum* malaria. Mechanisms that drive this protection remain unclear due to a poor understanding of malaria clinical phenotypes during infancy.

**Methods:**

We enrolled a birth cohort of 678 infants in Busia, Uganda, an area of high malaria transmission. We followed infants through 12 months of age and quantified protection against parasitemia and clinical disease.

**Results:**

Symptomatic malaria incidence increased from 1.2 to 2.6 episodes per person-year between 0 and <6 months and between 6 and 12 months of age, while the monthly probability of asymptomatic parasitemia given infection decreased from 32% to 21%. Sickle cell trait (HbAS) was protective against symptomatic malaria (incidence rate ratio  = 0.57 comparing HbAS vs hemoglobin AA (HbAA); 95% confidence interval, 0.44–0.74; *P* < .001), but age modified this relationship (P_int_ = <0.001), with nonlinear protection that waned between 0 and 9 months of age before increasing. Increasing age was associated with higher parasite densities at the time of infection and, in infants with HbAS, a reduced ability to tolerate high parasite densities without fever.

**Conclusions:**

Age-dependent changes in HbAS protective efficacy in infancy were accompanied by differential loss of antiparasite and antidisease protection among HbAS and HbAA infants. This provides a framework for investigating the mechanisms that underlie infant protection against malaria.

**Clinical Trials Registration:**

NCT02793622.

## Teaser

In 678 Ugandan infants followed through 12 months of age, 1131 malaria episodes were observed. Age-dependent changes in sickle cell trait (HbAS) protective efficacy were accompanied by differential loss of antiparasite and antidisease protection among HbAS and HbAA infants.

Malaria is responsible for 228 million clinical cases and 405 000 deaths annually, with morbidity and mortality highest in young African children due to *Plasmodium falciparum* [1]. Infants who live in highly endemic areas are thought to be partially protected against symptomatic malaria [2–4], though studies assessing the burden of malaria during the first year of life have been limited [5]. An improved understanding of antimalarial protection in infancy may motivate novel interventions to prevent adverse malaria outcomes in children.

Epidemiologic studies suggest that detection of microscopic parasitemia may be uncommon in the first 6 months of life [2]. This has partially been ascribed to reduced vector exposure (eg, swaddling), leading to a lower risk of infection [6]. However, once infected, infants also present with lower parasite densities [7] and asymptomatic infection [4, 8], findings that typically occur only after years of repeated infection [9]. Several potential mechanisms for protection against high-density parasitemia and symptomatic malaria in infants have been proposed, including the presence of fetal hemoglobin (HbF), which may be less susceptible to *Plasmodium* infection [10, 11], and maternally acquired antibodies [12], although definitive evidence for these mechanisms has proven elusive. One reason it has been challenging to define protective mechanisms in infants is that few available birth cohorts have independently detailed the risk of infection and the risk of disease once infected [9, 13]. Furthermore, it is unclear whether infant characteristics, such as sickle cell trait [14–17], modify these risks.

To better understand malaria clinical phenotypes among infants, we followed a birth cohort of infants living in a high transmission setting in southeastern Uganda. We quantified age-related changes in malaria incidence and the probability of symptoms given infection in infancy and determined the relative protective effects of both individual and household-level factors. We then focused on 2 specific types of protection: antiparasite (ie, the ability to control parasite densities upon infection) and antidisease (the ability to tolerate higher parasite densities without developing fever), as these have been defined as independent components of acquired antimalarial immunity [9, 18].

## METHODS

### Study Setting and Participants

This study was conducted from September 2016 to December 2018 in Busia district, Uganda, an area of high malaria transmission intensity. Liveborn infants included in this study were born to women enrolled in a trial of intermittent preventive treatment of malaria in pregnancy (IPTp); inclusion and exclusion criteria for the parent trial have been published [19]. Briefly, pregnant women were enrolled between 12 and 20 weeks of gestation, randomized to receive IPTp with monthly sulfadoxine pyrimethamine (SP) or monthly dihydroartemisinin piperaquine (DP), and followed through delivery. The study was approved by ethics committees at Makerere University School of Biomedical Sciences, University of California San Francisco, and Stanford University.

### Study Procedures

All women were provided long-lasting insecticide-treated bednets at enrollment, and a household survey was conducted to collect socioeconomic and house construction data [20]. Handheld global positioning system navigators (GARMIN eTrex, Olathe, KS) were used to record coordinates of participants’ homes, and ArcGIS was used for map projection.

Mothers were encouraged to bring their infants to a study clinic for all care. Routine assessments were conducted every 4 weeks, including collection of blood for detection of malaria parasites. Infants who presented with a history of fever in the past 24 hours or were found to have a tympanic temperature ≥38.0°C on clinical assessment had a blood smear performed for detection of malaria parasites. If the smear was positive, infants were treated for malaria according to the Uganda Ministry of Health guidelines. For uncomplicated malaria, infants were treated with artemether-lumefantrine. For complicated malaria (malaria with danger signs) and severe malaria, infants were treated with intravenous artesunate. Asymptomatic parasitemia was not treated, and children diagnosed with sickle cell disease were provided weekly chloroquine prophylaxis, per Ugandan guidelines. Infants were followed to aged 12 months. Criteria for premature study withdrawal included movement out of the study area, inability to be located for >60 days, withdrawal of consent, and death.

### Laboratory Procedures

Blood smears were stained with 2% Giemsa and read by experienced microscopists. Blood smears were considered negative when the examination of 100 high-power fields did not reveal asexual parasites. All slides were read by a second microscopist, and a third reader settled any discrepancies. Hemoglobin (Hb) genotype was ascertained by polymerase chain reaction (PCR)–based detection [21] using DNA extracted from dried blood spots collected during clinic visits.

### Statistical Analyses

Analyses were conducted using Stata version 14 and R version 3.6.1. Incident outcomes included symptomatic malaria (malaria episode that required treatment and not preceded by another episode in the prior 14 days) and complicated malaria (malaria with danger signs or severe malaria). For monthly prevalence measures, we considered a 28-day window around each routine visit for the presence/absence of malaria parasitemia. If parasitemic, monthly periods were further characterized as symptomatic malaria (from 21 days prior to 7 days after the routine visit) or asymptomatic parasitemia (positive routine blood smear in the absence of fever, without symptomatic malaria 21 days prior to 7 days after the visit). We also considered the peak parasite density and objective temperature measured while parasitemic during this 4-week window.

Exposure variables included infant characteristics at birth (sex, birth weight, gestational age at delivery, and Hb genotype) and household characteristics (household wealth, maternal education level, distance from clinic, and housing construction). Principal components analysis was used to generate a wealth index based on ownership of common household items [20], with households grouped into tertiles. Distance from clinic was categorized as households being <5 or ≥5 km from the study clinic. Housing construction types were classified as modern (plaster or cement walls, metal or wooden roofs, and closed eaves) or traditional (all other houses) [22].

Follow-up started at birth and ended on the day the infant was aged 12 months or prematurely withdrawn. Incident outcomes were compared using negative binomial regression models. Repeated prevalence measures were compared using log-binomial, log-Poisson, or Gaussian models with robust standard errors and generalized estimating equations to adjust for clustering. Exposure variables found to be significant on univariate analyses were included in multivariate models; these models also included maternal IPTp regimen. We assessed for interaction between exposure variables and age. In all analyses, 2-tailed *P* values  < .05 were considered statistically significant.

Generalized additive models were used to model and visualize associations between age, parasite density, and probability of symptoms if infected (Supplementary Methods). For antiparasite protection, the outcome was the parasite density recorded at each parasite-positive study visit [9]. For antidisease protection, the outcome was the objective temperature recorded during parasite-positive visits, conditional on the parasite density.

## RESULTS

### Study Participants and Household Characteristics

**Figure 1.**
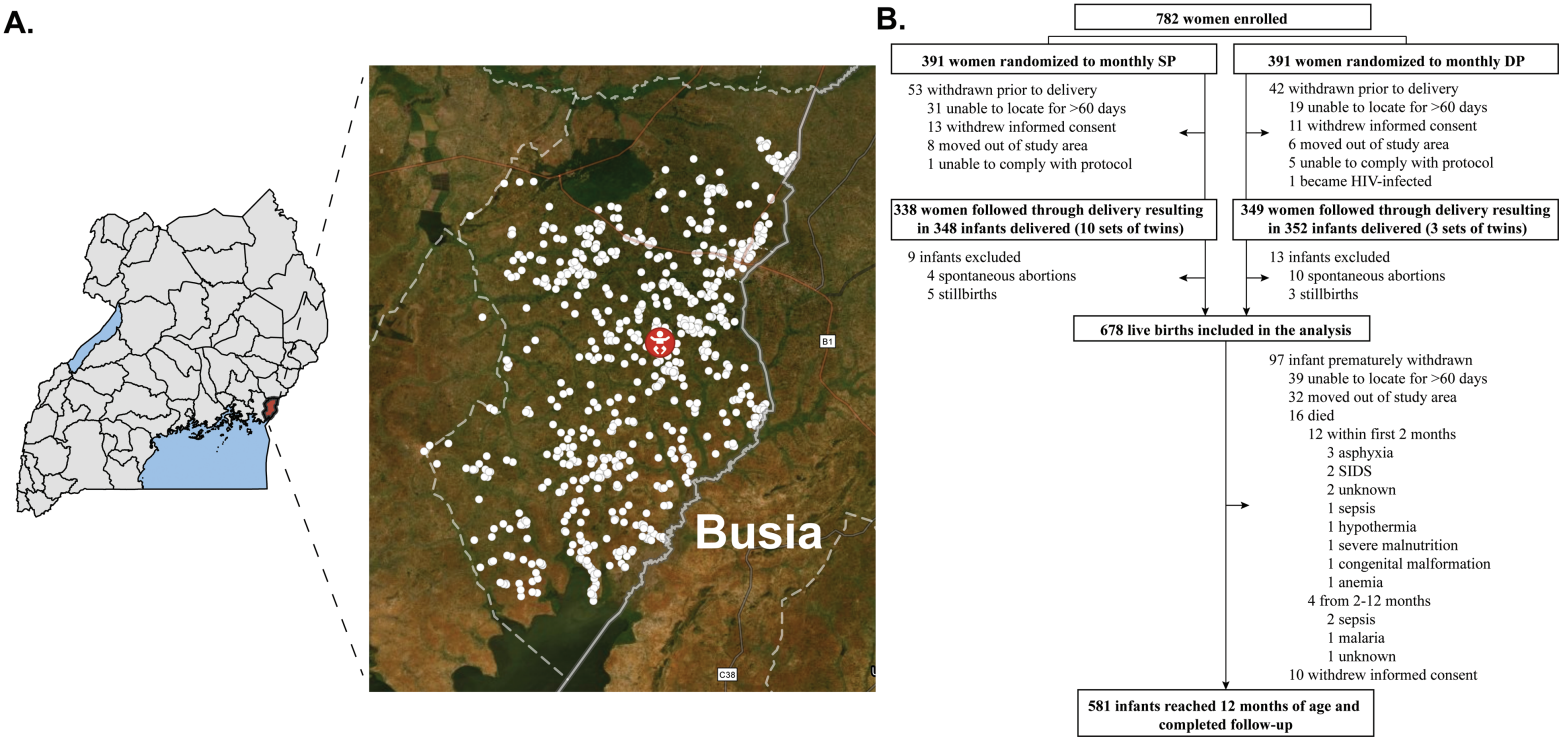
Study site and profile. *A,* Distribution of households in Busia District, Uganda. *B,* Trial profile. Abbreviations: DP, dihydroartemisinin piperaquine; HIV, human immunodeficiency virus; SIDS, sudden infant death syndrome; SP, sulfadoxine pyrimethamine.

Between December 2016 and December 2017, 678 live infants were born to 666 mothers. Maternal households ranged from 0.1 to 26 km from the study clinic (Figure 1A). The majority of mothers (76.6%) had a primary level education or less, and most households (77.5%) were built with traditional materials (Table 1).

**Table 1.** Baseline Characteristics and Descriptive Statistics Through 1 Year of Life

Characteristic	Overall	HbAA	HbAS (With Sickle Cell Trait)	HbSS (With Sickle Cell Disease)
Mothers and households^a^	n = 666	n = 507	n = 119	n = 13
Maternal sulfadoxine-pyrimethamine intermittent preventive treatment of malaria in pregnancy	330 (49.6%)	253 (49.9%)	61 (51.3%)	4 (30.8%)
Maternal education, primary education or less	510 (76.6%)	395 (77.9%)	82 (68.9%)	11 (84.6%)
Traditional housing construction	516 (77.5%)	397 (78.3%)	88 (74%)	7 (53.9%)
House greater than 5 km from the clinic, n/N (%)	521/658 (79.2)	396 (78.9)	93 (80.2)	8 (61.5)
Children at birth^a^	n = 678	n = 515	n = 122	n = 13
Female sex, n (%)	346 (51.0)	271 (52.6)	57 (46.7)	4 (30.8)
Gestational age at birth, mean (range), weeks	39.5 (28.4–43.9)	39.5 (29.6–43)	39.4 (28.4–43.9)	39.3 (35.4–41.4)
Preterm births (<37 weeks of gestation), n (%)	44 (6.5)	29 (5.6)	9 (7.4)	2 (15.4)
Birth weight, mean (range), g	3037 (1100–4230)	3043 (1380–4230)	3059 (1100–4100)	3105 (2360–3740)
Low birth weight (<2500 g), n (%)	60 (8.9)	39 (7.6)	12 (9.8)	1 (7.7)
Incident outcomes through 1 year of life				
Total incident episodes of malaria	1131	986	127	7
Uncomplicated malaria treated with artemether lumefantrine	1066	925	124	6
Complicated malaria (malaria with danger signs)	57	53	3	1
Severe malaria	8	8	0	0
Symptomatic malaria incidence through 1 year of life (range), ppy	1.84 (0–10)	2.04 (0–10)	1.17 (0–10)	0.58 (0–2)
Complicated malaria incidence through 1 year of life (range), ppy	0.11 (0–3)	0.11 (0–3)	0.03 (0–1)	0.08 (0–1)
Severe malaria incidence through 1 year of life (range), ppy	0.01 (0–1)	0.02 (0–1)	0	0
Total hospitalizations	27	21	2	1
Total deaths	16	6	2	0
Prevalence outcomes				
Use of long-lasting insecticidal net night prior to routine visit^b^	7803/7829 (99.7%)	6160/6182 (99.6%)	1379/1383 (99.7%)	158/158 (100%)
Microscopic parasite prevalence (4-week intervals), n/N (%)	1518/7829 (19.4)	1264/6182 (20.4)	229/1383 (16.6)	12/158 (7.6)
Asymptomatic if parasitemic (4-week intervals), n/N (%)	392/1518 (25.8)	289/1264 (22.9)	96/229 (41.9)	5/12 (41.7)

Abbreviations: HbAA, hemoglobin AA; ppy, per person-years.

^a^Hemoglobin genotype not ascertained in 28 infants living in 27 maternal households.

^b^Long-lasting insecticidal net compliance was ascertained through self-report at each participant visit where parents/guardians were asked if child slept under a bednet the previous night.

Hb genotype was ascertained in 650 of 678 (95.5%) infants with available DNA for testing, of which 79.2% were HbAA, 18.8% sickle cell trait (HbAS), and 2.0% HbSS (with sickle cell disease). Pediatric characteristics at birth were similar between Hb genotype groups (Table 1). Of 97 infants prematurely withdrawn before age 1 year, 39 were unable to be located for >60 days, 32 moved out of the study area, 16 died, and 10 withdrew consent. Of 16 deaths, 12 occurred within the first 2 months of life, with asphyxia (n = 3; Figure 1B) and sepsis (n = 3) the most common causes of death.

### Symptomatic Malaria in Infancy

**Figure 2.**
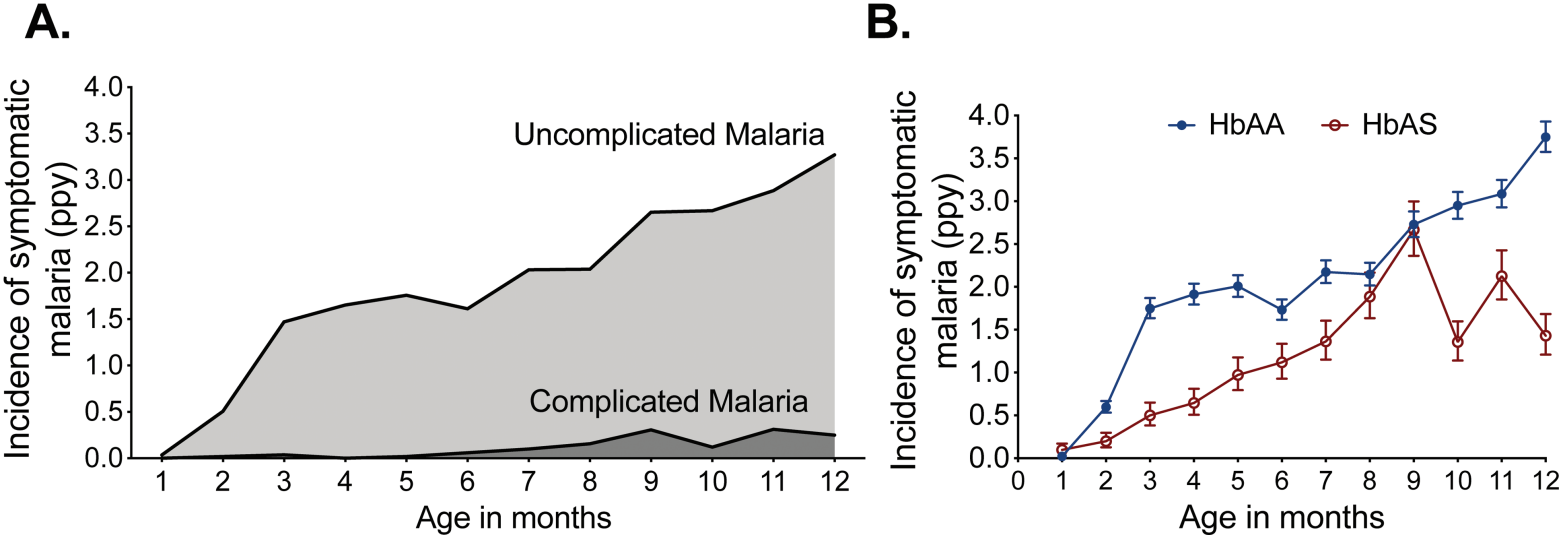
Incidence of symptomatic malaria in the first year of life and effect modification by sickle cell trait. *A,* Overall incidence of uncomplicated and complicated malaria by age. *B,* Incidence of symptomatic malaria by age stratified by hemoglobin genotype. Error bars represent 95% confidence intervals. Abbreviation: ppy, per person-years.

Overall, 1131 incident episodes of symptomatic malaria were observed; incidence increased from 1.15 episodes per person-year (ppy) between 0 and <6 months of age to 2.58 episodes ppy between 6 and 12 months of age (Figure 2A). Infants with HbAS had 39% less symptomatic malaria than HbAA infants (Table 2, Supplementary Figure 1), but we observed nonlinear interaction between Hb genotype and age (P_int_ = 0.001; Figure 2B). Infants with HbAS had 66% less symptomatic malaria between 0 and 3 months (incidence rate ratio [IRR], 0.34; 95% confidence interval [CI], .17–.68; *P* = .002) and 51% less symptomatic malaria between 3 and 6 months of age (IRR, 0.49; 95% CI, .31–.77; *P* = .002) compared with HbAA infants. Between 6 and 9 months of age, there was no significant difference between HbAS vs HbAA infants (IRR, 0.84; 95% CI, .62–1.15; *P* = .27). From 9 to 12 months of age, infants with HbAS again had 49% less symptomatic malaria than HbAA infants (IRR, 0.51; 95% CI, .37–.69; *P* < .001). Infants with sickle cell disease (HbSS) had 69% less symptomatic malaria than HbAA infants (adjusted IRR [aIRR], 0.31; 95% CI, .16–.61; *P* = .001) without significant interaction with age (P_int_ = 0.26), although this may have been due (in part) to these infants receiving chloroquine prophylaxis, given evidence of a return of parasite susceptibility to chloroquine in Uganda [23]. Other infant characteristics were not associated with symptomatic malaria incidence (Table 2).

**Table 2.** Factors Associated With Incidence of Malaria

Risk Factor	Category	Number of Children	Episodes of Malaria	Person-Years of Observation	Incidence of Malaria Per Person-Years	Incidence Rate Ratio (95% CI)	*P* Value	Adjusted Incidence Rate Ratio^a^ (95% CI)	*P* Value
Age, months	0 to 6		366	317.4	1.15	Ref		Ref	
	>6 to 12		765	296.1	2.58	2.24 (2.00–2.52)	<.001	2.19 (1.95–2.45)	<.001
Season	Aug–Oct		193	157.2	1.23	Ref		Ref	
	Feb–Mar		156	97.0	1.61	1.31 (1.07–1.60)	.009	1.00 (0.82–1.22)	.99
	Nov–Jan		286	152.7	1.87	1.53 (1.30–1.80)	<.001	1.30 (1.10–1.53)	.002
	Apr–Jul		496	206.6	2.40	1.96 (1.68–2.28)	<.001	1.70 (1.45–1.98)	<.001
Infant hemoglobin genotype^**b**^	HbAA	515	986	483.7	2.04	Ref		Ref	
	HbAS (with sickle cell trait)	122	127	108.8	1.17	0.57 (0.44–0.74)	<.001	0.61 (0.48–0.79)	<.001
	HbSS (with sickle cell disease)	13	7	12.1	0.58	0.28 (0.15–0.53)	<.001	0.31 (0.16–0.58)	.001
Infant sex	Male	332	545	296.2	1.84	Ref		Ref	
	Female	346	586	317.4	1.85	1.01 (0.85–1.21)	.88		
Preterm birth	No	634	1075	578.7	1.86	Ref		Ref	
	Yes	44	56	34.9	1.60	0.85 (0.58–1.25)	.40		
Low birth weight	No	618	1059	567.6	1.87	Ref		Ref	
	Yes	60	72	45.9	1.57	0.83 (0.60–1.17)	.30		
Maternal intermittent preventive treatment of malaria in pregnancy	Sulfadoxine pyrimethamine	339	602	304.4	1.98	Ref		Ref	
	Dihydroartemisinin piperaquine	339	529	309.2	1.71	0.87 (0.73–1.03)	.10		
Education level	None or primary	520	960	473.0	2.03	Ref		Ref	
	O level or higher	158	171	140.6	1.22	0.59 (0.47–0.75)	<.001	0.62 (0.49–0.78)	<.001
Housing type	Traditional	525	939	472.2	1.99	Ref		Ref	
	Modern	153	192	141.3	1.36	0.68 (0.54–0.86)	.002	0.73 (0.58–0.92)	.007
Household wealth	Poorest	236	405	208.6	1.94	Ref		Ref	
	Mid	223	386	203.4	1.90	0.98 (0.80–1.21)	.85		
	Highest	219	340	201.5	1.69	0.87 (0.70–1.08)	.21		
Distance from clinic^**c**^	≥5 km	528	889	473.3	1.88	Ref		Ref	
	<5 km	142	230	132.2	1.74	0.93 (0.75–1.15)	.50		

Abbreviations: CI, confidence interval; HbAA, hemoglobin AA.

^a^Adjusted incidence rate ratio, with adjustment for age, season, infant hemoglobin (Hb) genotype, maternal education, and household construction type.

^b^N = 650 with Hb genotype.

^c^n = 670 with household distance measured.

Regarding maternal and household characteristics, infants born to mothers with O level of education or higher had 38% less symptomatic malaria than infants born to mothers with none or primary level education (aIRR, 0.62; 95% CI, .48–.78; *P* < .001). Infants living in houses constructed with modern materials had 27% less symptomatic malaria than infants living in traditional households (aIRR, 0.73; 95% CI, .58–.92; *P* = .008). No significant interactions were observed between infant age, symptomatic malaria, and maternal education (P_int_ = 0.92) or house type (P_int_ = 0.40).

### Complicated Malaria in Infancy

There were 65 incident episodes of complicated malaria (0.11 episodes ppy; Table 1), 57 episodes of malaria with danger signs (47 severe emesis, 6 inability to breastfeed, 2 convulsions, 2 lethargy), and 8 episodes of severe malaria (7 respiratory distress, 1 severe anemia). All cases of severe malaria were among HbAA infants, and no child had severe malaria more than once. One case of severe malaria resulted in the death of a child, a 9-month-old who presented with fever, multiple seizures, respiratory distress, a parasite density of approximately 150 000 parasites/μL, and Hb of 6.4 gm/dL. She died a day after presentation despite being hospitalized and treated with intravenous artesunate and antibiotics. This was the child’s third episode of malaria; her previous 2 episodes were uncomplicated.

The incidence of complicated malaria was nearly 7 times higher between 6 and 12 months of age compared with 0 and 6 months of age (aIRR, 6.81; 95% CI, 3.23–14.4; *P* < .001; Figure 2A). Infants with HbAS had 83% less complicated malaria compared with HbAA infants (aIRR, 0.17; 95% CI, .04–.68; *P* = .01), with no significant interaction observed between age and Hb genotype (P_int_ = 0.27). Other infant characteristics were not significantly associated with the incidence of complicated malaria (Table 3).

**Table 3.** Factors Associated With Incidence of Complicated Malaria

Risk Factor	Category	Number of Children	Episodes of Complicated Malaria	Person-Years of Observation	Incidence Per Person-Years	Incidence Rate Ratio (95% CI)	*P* Value	Adjusted Incidence Rate Ratio^a^ (95% CI)	*P* Value
Age, months	0 to 6		7	317.4	0.02	Ref		Ref	
	>6 to 12		58	296.1	0.20	8.89 (4.11–19.2)	<.001	6.81 (3.23–14.4)	<.001
Season	Aug–Oct		6	157.2	0.04	Ref		Ref	
	Feb–Mar		21	97.0	0.22	5.68 (2.24–14.4)	<.001	4.47 (1.54–13.0)	.006
	Nov–Jan		7	152.7	0.05	1.20 (0.44–3.29)	.72	1.21 (0.39–3.78)	.74
	Apr–Jul		31	206.6	0.15	3.94 (1.71–9.09)	.001	3.95 (1.50–10.4)	.005
Infant hemoglobin genotype^**b**^	HbAA	515	60	483.7	0.12	Ref		Ref	
	HbAS (with sickle cell trait)	122	3	108.8	0.03	0.22 (0.07–0.70)	.01	0.17 (0.04–0.68)	.01
	HbSS (with sickle cell disease)	13	1	12.1	0.08	0.67 (0.10–4.44)	.67	0.83 (0.12–5.59)	.85
Infant sex	Male	332	38	296.2	0.13	Ref		Ref	
	Female	346	27	317.4	0.09	0.66 (0.39–1.12)	.13		
Preterm birth	No	634	63	578.7	0.11	Ref		Ref	
	Yes	44	2	34.9	0.06	0.53 (0.13–2.08)	.36		
Low birth weight	No	618	64	567.6	0.11	Ref		Ref	
	Yes	60	1	45.9	0.02	0.19 (0.03–1.37)	.10		
Maternal intermittent preventive treatment of malaria in pregnancy	Sulfadoxine pyrimethamine	339	42	304.4	0.14	Ref		Ref	
	Dihydroartemisinin piperaquine	339	23	309.2	0.07	0.54 (0.31–0.93)	.03	0.56 (0.33–0.95)	.03
Education level	None or primary	520	57	473.0	0.12	Ref		Ref	
	O level or higher	158	8	140.6	0.06	0.47 (0.18–1.21)	.12		
Housing type	Traditional	525	52	472.2	0.11	Ref		Ref	
	Modern	153	13	141.3	0.09	0.84 (0.42–1.68)	.61		
Household wealth	Poorest	236	29	208.6	0.14	Ref		Ref	
	Mid	223	28	203.4	0.14	0.99 (0.57–1.73)	.97	0.93 (0.52–1.65)	.80
	Highest	219	8	201.5	0.04	0.29 (0.13–0.64)	.002	0.29 (0.13–0.65)	.003
Distance from clinic^**c**^	≥5 km	528	57	473.3	0.12	Ref		Ref	
	<5 km	142	7	132.2	0.05	0.44 (0.20–0.95)	.04	0.43 (0.20–0.93)	.03

Abbreviations: CI, confidence interval; HbAA, hemoglobin AA.

^a^Adjusted incidence rate ratio, with adjustment for age, season, infant hemoglobin (Hb) genotype, maternal intermittent preventive treatment of malaria in pregnancy, household wealth, and distance from clinic.

^b^N = 650 with Hb genotype.

^c^n = 670 with household distance measured.

Infants whose mothers were given IPTp with DP had 44% less complicated malaria than infants whose mothers were given IPTp with SP (Table 3), as previously reported [24]. Infants living in the wealthiest households had 71% less complicated malaria compared with those living in the poorest households (aIRR, 0.29; 95% CI, .13–.64; *P* = .002). Infants living closer (<5 km) to the study clinic had 57% less complicated malaria compared with those living ≥ 5km from the clinic (aIRR, 0.43; 95% CI, .20–.93; *P* = .03). Neither maternal education nor house type were associated with protection against complicated malaria (Table 3).

### Parasite Prevalence and Probability That Parasitemia Is Asymptomatic in Infancy

**Figure 3.**
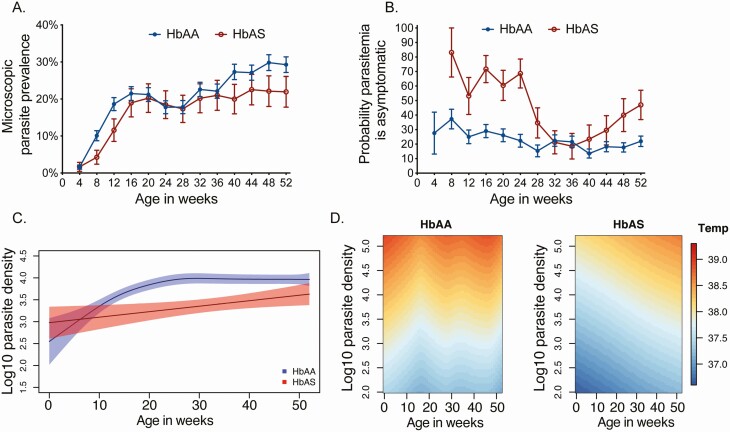
Probability infection is asymptomatic in infancy is modified by sickle cell trait. *A,* Parasite prevalence stratified by hemoglobin (Hb) genotype. *B,* Probability of asymptomatic parasitemia given infection stratified by Hb genotype. In *A* and *B*, error bars represent standard errors estimated using generalized estimating equations. *C,* Mean log_10_ parasite densities given an infection during the first year of life stratified by Hb genotype. Shaded area represents 95% confidence intervals. *D,* Temperature at a given parasite density during the first year of life stratified by Hb genotype (hemoglobin AA [HbAA], left; HbAS [with sickle cell trait], right.)

Microscopic parasite prevalence, measured every 4 weeks and including both symptomatic and asymptomatic infections, increased from 14.3% between 0 and 6 months of age to 23.7% between 6 and 12 months of age. Parasite prevalence did not differ significantly by Hb genotype overall (prevalence rate ratio [PRR], 0.84; 95% CI, .69–1.03; *P* = .10), without significant interaction with age (P_int_ = 0.20; Figure 3A, Supplementary Table 1).

The probability that parasitemia was asymptomatic decreased from 32.1% between 0 and 6 months of age to 20.7% between 6 and 12 months of age (aPRR, 0.65; 95% CI, .54–.768; *P* < .001) and was significantly higher in infants with HbAS (Supplementary Table 2). However, we observed nonlinear interactions between Hb genotype, the probability of asymptomatic parasitemia given infection, and age (P_int_ = 0.04; Figure 3B). From 0 to <6 months of age, infants with HbAS had approximately 2-fold higher probability that parasitemia was asymptomatic than HbAA infants (Table 4). From 6 to 9 months of age, there was no difference in the probability that parasitemia was asymptomatic (PRR, 1.07; 95% CI, .62–1.85; *P* = .81); however, from 9 to 12 months of age, infants with HbAS again had a significantly higher probability that parasitemia was asymptomatic (PRR, 1.89; 95% CI, 1.32–2.71; *P* < .001).

**Table 4.** Effect Modifications Between Sickle Cell Trait, Age, and Clinical Phenotypes of Malaria in Infancy

	Malaria Incidence				Prevalence Asymptomatic Parasitemia Given Infection^a^			
Age, Months	Episodes (Incidence Per Person-Years)		Incidence Rate Ratio (95% CI)	*P* Value	n/N (%)		Prevalence Rate Ratio (95% CI)	*P* Value
	HbAS (With Sickle Cell Trait)	HbAA (Ref)			HbAS (With Sickle Cell Trait)	HbAA (Ref)		
0 to <3	8 (0.27)	100 (0.78)	0.34 (0.17–0.68)	.002	11/22 (50.00)	45/160 (28.13)	1.79 (1.05–3.07)	.03
3 to <6	25 (0.92)	228 (1.87)	0.49 (0.31–0.77)	.002	42/62 (67.74)	84/290 (28.97)	2.42 (1.82–3.21)	<.001
6 to <9	52 (1.98)	279 (2.36)	0.84 (0.62–1.15)	.27	14/61 (22.95)	63/290 (21.72)	1.07 (0.62–1.85)	.81
9 to <12	42 (1.65)	379 (3.27)	0.51 (0.37–0.69)	<.001	29/84 (34.52)	97/524 (18.51)	1.89 (1.32–2.70)	<.001
	**Parasite Densities at Time of Infection (Antiparasite Protection)**							
**Age, months**	**HbAS**				**HbAA**			
	**Log**_**10**_ **Parasite Density, Mean (SE)**	**Coefficient (95% CI)** ^b^	***P* Value**		**Log**_**10**_ **Parasite Density, Mean (SE)**	**Coefficient (95% CI)** ^b^	***P* Value**	
0 to <3	3.27 (0.18)	Ref			3.40 (0.08)	Ref		
3 to <6	3.13 (0.14)	–0.14 (≠0.57–0.29)	.52		3.86 (0.06)	0.46 (0.28–0.65)	<.001	
6 to <9	3.60 (0.12)	0.32 (00.10–0.75)	.14		4.05 (0.06)	0.65 (0.46–0.85)	<.001	
9 to <12	3.59 (0.13)	0.31 (–0.13–0.75)	.16		4.09 (0.05)	0.70 (0.52–0.88)	<.001	
	**Temperature Experienced at Parasite Densities >10** ^**4**^ **Parasites/μL (Antidisease Protection)**							
**Age, months**	**HbAS**				**HbAA**			
	**Temp, Mean (SE)**	**Coefficient (95% CI)** ^b^	***P* Value**		**Temperature, Mean (SE)**	**Coefficient (95% CI)** ^b^	***P* Value**	
0 to <3	36.89 (0.09)	Ref			38.42 (0.12)	Ref		
3 to <6	37.99 (0.26)	1.10 (0.54–1.66)	<.001		38.30 (0.10)	–0.13 (–0.42–0.17)	.41	
6 to <9	38.32 (0.23)	1.43 (0.93–1.94)	<.001		38.51 (0.09)	0.09 (–0.18–0.35)	.53	
9 to <12	38.61 (0.22)	1.73 (1.27–2.19)	<.001		38.63 (0.08)	0.21 (–0.05–0.46)	.11	

Abbreviations: CI, confidence interval; HbAA, hemoglobin AA; SE, standard error.

^a^Prevalence asymptomatic parasitemia given microscopic infection, as measured during 4-week windows. ^b^Coefficients estimated using generalized estimating equations with robust standard errors.

### Antiparasite and Antidisease Protection in Infancy

Finally, we sought to determine whether the rising incidence of symptomatic malaria and decline in asymptomatic parasitemia were due to loss of the ability to control parasite densities (ie, antiparasite protection) vs loss of the ability to tolerate higher parasite densities without fever (ie, antidisease protection).

Among infants with HbAA, mean parasite densities at the time of any infection increased from 3.4 log_10_ parasites/μL from 0 to <3 months of age to 4.1 log_10_ parasites/μL from 6 to <12 months of age (Table 4, Figure 3C, Supplementary Figure 3). In contrast, mean temperatures experienced by HbAA infants at parasite densities >10^4^ parasites/μL was >38°C across infancy and did not significantly change with age (Table 4, Figure 3D). This suggests that among HbAA infants, increasing age was associated with loss of antiparasite protection.

Among infants with HbAS, mean parasite densities at the time of any infection also increased modestly with age, from 3.3 log_10_ parasites/μL at 0 to 3 months of age to 3.6 log_10_ parasites/μL at 6 to 12 months of age (Table 4, Figure 3C, Supplementary Figure 3). However, the temperature experienced by HbAS infants at parasite densities >10^4^ parasites/μL rose significantly during the first year of life, from <38.0°C at 0 to <6 months of age to >38°C at 6 to <12 months of age (Table 4, Figure 3D). These data suggest that among infants with HbAS, increasing age was associated with loss of predominantly antidisease protection.

## Discussion

In this birth cohort, the incidence of symptomatic malaria in infancy was high, rising with age, with the vast majority of cases uncomplicated. HbAS genotype was protective against symptomatic malaria in infancy, but age modified this relationship, with a nonlinear protective effect that waned between 0 and 9 months of age before increasing. Infants with HbAS had a significantly higher probability of asymptomatic parasitemia given infection than HbAA infants, but this was also similarly modified by age. Surprisingly, although increasing age was associated with higher parasite densities in all infants, infants with HbAS had a reduced ability to tolerate high parasite densities.

Consistent with prior reports, infants with HbAS had 40% less symptomatic malaria and >80% less complicated malaria than HbAA infants [7, 17]. However, to our knowledge, this is the first study to suggest that HbAS protection against symptomatic malaria wanes in the first 9 months of life before increasing. Mechanisms of protection afforded by HbAS genotype are likely multifactorial and include hypoxic inhibition of parasite growth [25, 26], superior clearance of infected red blood cells in the spleen [27–29], increased expression of heme oxygenase-1 [30], and Hemoglobin S polymerization-dependent parasite growth inhibition [31]. Furthermore, protection afforded by HbAS genotype increases with age, suggesting an interaction between HbAS genotype and acquired antimalarial immunity [32–34]. Given the loss of antidisease protection observed in infants with HbAS, we hypothesize that there likely exists synergisms between HbAS genotype, maternally acquired antimalarial antibodies, and clearance of parasites [35] that decline in the setting of waning maternal titers. We speculate that increasing HbAS protection later in infancy may be driven by acquisition of infant antimalarial antibodies [32], although this remains to be determined.

Parasite densities at the time of infection rose among all infants, suggesting waning antiparasite protection in infancy. Although mechanisms that drive this loss of antiparasite protection are unclear, HbF, which is present early in life, has been thought to be protective against *Plasmodium* infection [10, 11, 36]. However, a more recent study found that *Plasmodium* can develop normally in HbF erythrocytes [35]. Many have also hypothesized a role for maternally derived antibodies in protection against parasitemia in infants. However, others have instead reported that certain malaria-specific antibodies are associated with an increased risk of infection [37, 38], suggesting that antibodies may alternatively be markers of exposure. More systematic investigation of the role of maternally derived antibodies in antiparasite protection in infancy is needed.

Importantly, modifiable factors including living within a house constructed of modern materials and having a mother with higher educational achievement were associated with protection against symptomatic malaria, and higher household wealth and closer proximity to the study clinic were protective against complicated malaria. The protection afforded by these modifiable factors was similar in magnitude to sickle cell trait and that potentially offered by the RTS,S/AS02A vaccine [39]. The vast majority of malaria episodes in infants were uncomplicated, with <1% experiencing severe malaria. In a similar study conducted in Tanzania, 11.1% (102 of 882) experienced at least 1 episode of severe malaria, despite a similar prevalence of HbAS genotype [7]. Differences in the burden of severe malaria between the 2 studies may be due to the Tanzanian study being conducted before widespread implementation of long-lasting insecticidal nets and artemisinin-based combination therapies and smaller catchment area (731 km^2^ vs 1498 km^2^ in the Tanzanian study [7]), which may have reduced the time to initial treatment [40]. In the absence of a widely available malaria vaccine, public and private investments that address housing, education, and transportation are actionable and provide a range of benefits beyond malaria prevention.

Our study had limitations. We did not capture episodes of complicated and severe malaria occurring after age 1 year. However, prior reports suggest that the risk of severe malaria peaks within the first year of life [7]. Infants may have received medications outside the study clinic. However, self-reported use of outside medications was rare and unlikely to explain results stratified by age or by Hb genotype. Use of microscopy instead of PCR to diagnose infection may have underestimated the true prevalence of parasitemia. Unmeasured changes in HbF expression by age may have confounded our analyses. Finally, though we observed a relatively low burden of severe malaria and high prevalence of asymptomatic infection, these findings may not be generalizable to lower transmission settings.

By distinguishing between the risk of infection and risk of disease once infected and assessing interactions between age and sickle cell trait, our study provides a framework for investigating the mechanisms that underlie protection against malaria in infants. Defining these mechanisms, determine why they wane, and determine how they differ between infants with and without sickle cell trait may spur the development of novel vaccine and/or antibody-based preventive strategies for malaria.

## Supplementary Data

Supplementary materials are available at *Clinical Infectious Diseases* online. Consisting of data provided by the authors to benefit the reader, the posted materials are not copyedited and are the sole responsibility of the authors, so questions or comments should be addressed to the corresponding author.

ciab245_suppl_Supplementary_Materials

## Notes

***Acknowledgments.*** We thank the mothers and their infants who participated in the study, the administration and practitioners of Masafu General Hospital Busia, and staff members of the Infectious Diseases Research Collaboration.

***Disclaimer.*** The funders did not play a role in the study design, data collection, analysis, interpretation, and writing of the manuscript.

***Financial support.*** This work was supported by grants received from the Eunice Kennedy Shriver National Institute of Child Health and Human Development (grant P01 HD059454), the March of Dimes Foundation (Basil O’Connor Award to P. J.), and the Bill and Melinda Gates Foundation (OPP1141549).

***Potential conflicts of interest.*** N. Z. reports a Stanford Med Scholars Grant. All remaining authors: No reported conflicts of interest. All authors have submitted the ICMJE Form for Disclosure of Potential Conflicts of Interest. Conflicts that the editors consider relevant to the content of the manuscript have been disclosed.
