# Supplementary material for: Age-Related Changes in Malaria Clinical Phenotypes During Infancy Are Modified by Sickle Cell Trait
Source: Clin Infect Dis. 2021 Mar 19;73(10):1887–95. doi: 10.1093/cid/ciab245 (PMC8599196; doi:10.1093/cid/ciab245)
Supplement: ciab245_suppl_Supplementary_Materials [file ciab245_suppl_supplementary_materials.docx]

**Supplemental Material**

**Changing Clinical Phenotypes of Malaria in Infancy are Modified by Sickle Cell Trait**

Nicholas Zehner^1^, Harriet Adrama^2^, Abel Kakuru**^2^**, Teddy Andra^2^**,** Richard Kajubi**^2^**, Melissa Conrad**^3^**, Felistas Nankya**^2^**, Tamara D. Clark^3^, Moses Kamya^2,4^, Isabel Rodriguez-Barraquer^3^, Grant Dorsey^3^, Prasanna Jagannathan^1^

^1^Department of Medicine, Stanford University, Stanford, USA; ^2^Infectious Diseases Research Collaboration, Kampala, Uganda; ^3^Department of Medicine, University of California, San Francisco, USA; ^4^Department of Medicine, Makerere University College of Health Sciences, Kampala, Uganda

**Supplemental Methods:**

For anti-parasite protection, the outcome was the parasite density recorded at each parasite positive study visit. For anti-disease protection, the outcome was the objective temperature recorded during parasite positive visits, conditional on the parasite density.

The models were stratified by sickle cell status and fitted with the following form.

1. Anti-parasite protection

$${{Log}_{10}(Parasite density)}_{ijk}=f({age}_{ijk})$$

2. Anti- Disease protection

$${Temperature}_{ijk}=f({age}_{ijk}, {Log}_{10}Parasite density)$$

Where *i* is an index for individuals, *j* for households and *k* for specific visits. Thus, ${age}_{ijk}$ represents the age of child *i* from household *j* during visit *k.* Gams were fit in the R statistical framework using package mgcv (12). To account for clustering, all models included random effects at the individual and household levels.

**Supplementary Figure 1**


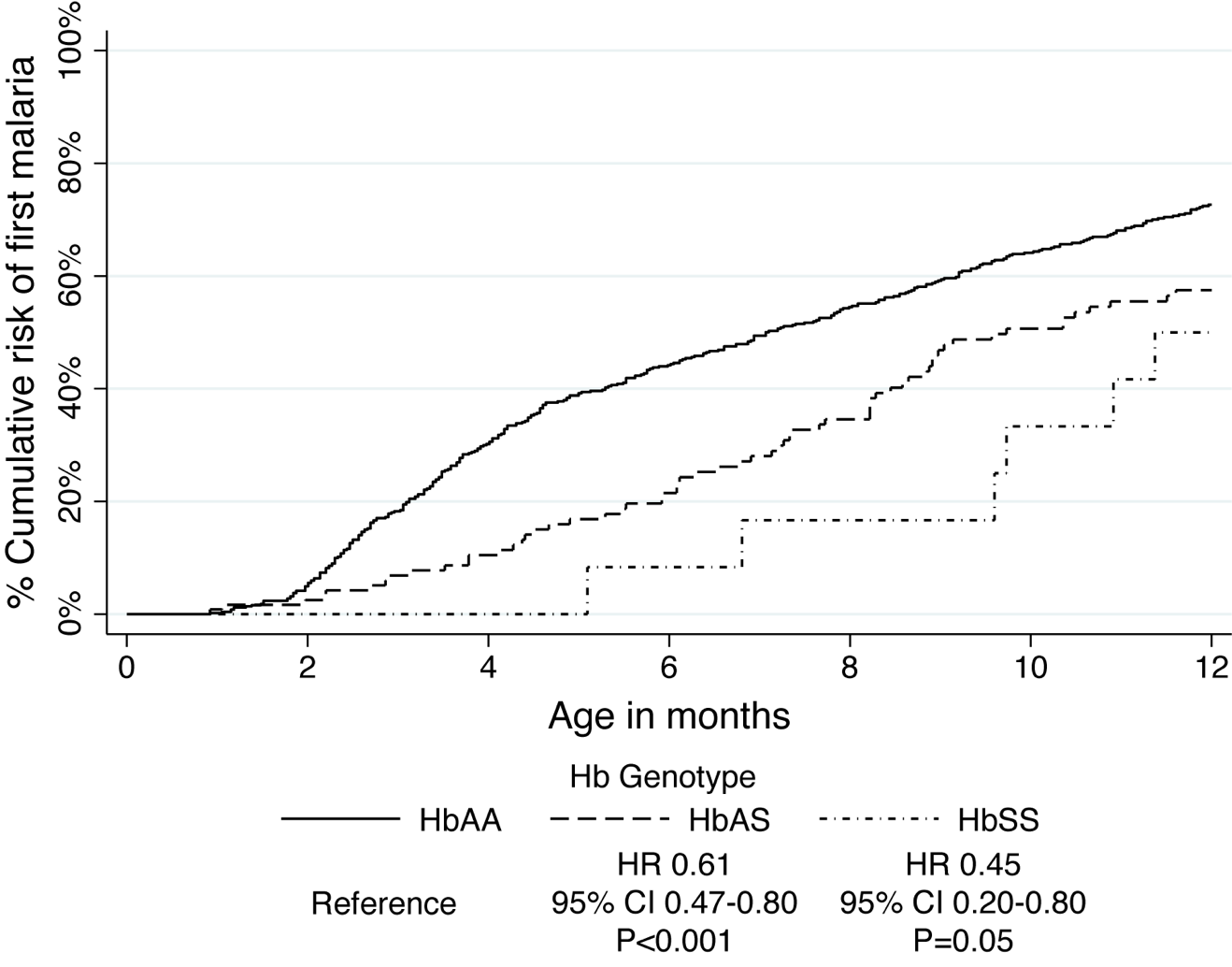


**Supplementary Figure 1: Cumulative risk of first malaria, stratified by Hb genotype.** The cumulative risk of any first episode of malaria was compared using the Kaplan–Meier product limit formula, and associations with Hb genotype assessed using a cox proportional hazards model.

**Supplementary Figure 2**


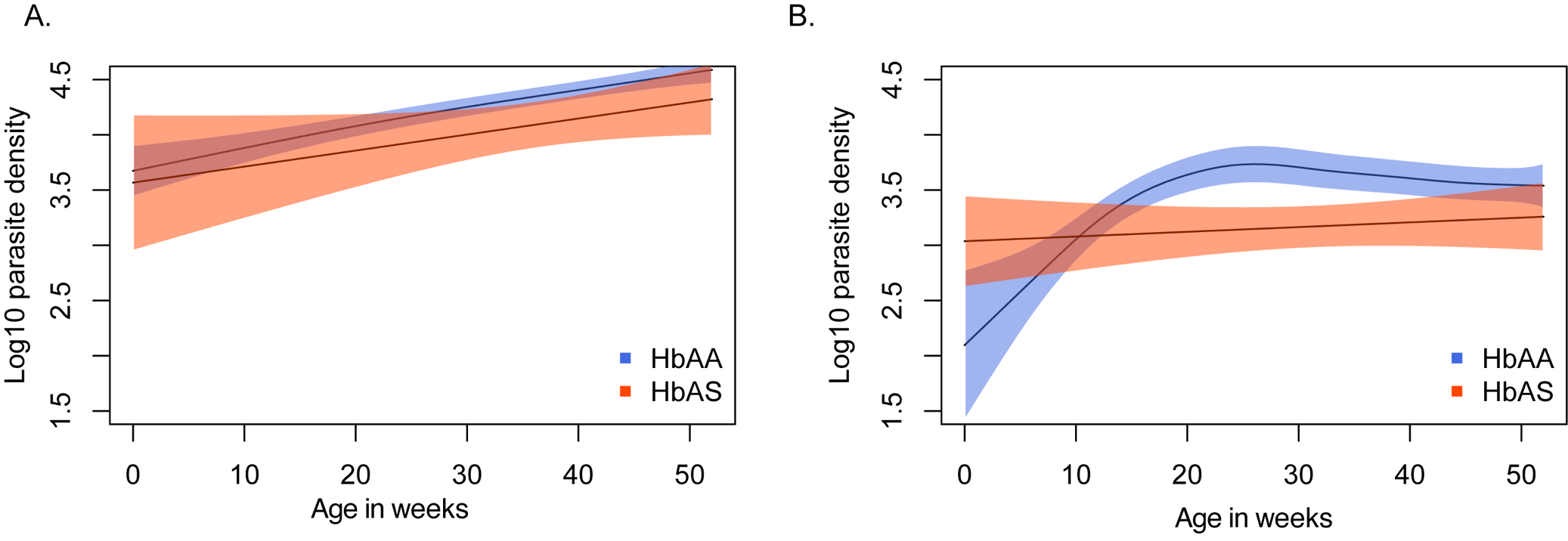


**Supplementary Figure 2.** Mean log parasite densities given an infection during the first year of life, stratified by Hb genotype, during symptomatic infections (A) and asymptomatic infections (B). Shaded areas represent 95% confidence intervals.

**Supplemental Table 1. Microscopic parasite prevalence, stratified by age in months and Hb genotype**

|  | **Microscopic parasite prevalence (probability of infection)** | | | | | |
| --- | --- | --- | --- | --- | --- | --- |
| **Age in months** | **n/N (%)** | | **PRR (95% CI)** | **p-value** | **aPRR^1^ (95% CI)** | **p-value** |
|  | **HbAS** | **HbAA (Ref)** |  |  |  |  |
| 0-<3 | 22/351 (6.3%) | 160/1517 (10.5%) | 0.59 (0.35-0.99) | 0.05 | 0.62 (0.38-1.03) | 0.06 |
| 3-<6 | 62/320 (19.4%) | 290/1436 (20.2%) | 0.96 (0.70-1.32) | 0.81 | 1.01 (0.74-1.37) | 0.96 |
| 6-<9 | 61/312 (19.6%) | 290/1392 (20.8%) | 0.93 (0.69-1.26) | 0.64 | 0.97 (0.72-1.30) | 0.83 |
| 9-<12 | 84/400 (21.0%) | 524/1837 (28.5%) | 0.74 (0.58-0.96) | 0.02 | 0.75 (0.58-0.96) | 0.02 |

PRR: Prevalence rate ratio

**^1^**Adjusted prevalence rate ratio, with adjustment for maternal IPTp arm, maternal education, season, household construction type, household wealth, and distance from clinic

**Supplemental Table 2. Factors associated with monthly probability of asymptomatic parasitemia given infection**

| **Risk Factor** | **Category** | **n/N (%)** | **PRR (95% CI)** | **p-value** | **aPRR^a^ (95% CI)** | **p-value** |
| --- | --- | --- | --- | --- | --- | --- |
| Age in months | 0-6 | 185/542 (34.1%) | reference |  | reference | |
|  | >6-12 | 207/976 (21.2%) | 0.65 (0.54-0.76) | <0.001 | 0.63 (0.48-0.83) | <0.001 |
| Season | Aug-Oct | 89/287 (31.0%) | reference |  | reference | |
|  | Feb-Mar | 62/226 (27.4%) | 0.93 (0.70-1.21) | 0.58 | 1.07 (0.82-1.41) | 0.62 |
|  | Nov-Jan | 97/369 (26.3%) | 0.90 (0.71-1.14) | 0.38 | 0.96 (0.76-1.22) | 0.71 |
|  | Apr-Jul | 144/636 (22.6%) | 0.79 (0.63-0.98) | 0.03 | 0.85 (0.68-1.07) | 0.16 |
| Infant Hb genotype^b^ | HbAA | 289/1264 (22.9%) | reference |  | reference | |
|  | HbAS | 96/229 (41.9%) | 1.98 (1.58-2.47) | 0.01 | 2.00 (1.60-2.47) | <0.001 |
|  | HbSS | 5/12 (41.7%) | 1.49 (0.61-3.62) | 0.38 | 1.65 (0.80-3.41) | 0.18 |
| Infant Sex | Male | 184/729 (25.2%) | reference | | reference | |
|  | Female | 208/789 (26.4%) | 1.01 (0.81-1.26) | 0.95 |  |  |
| Preterm birth | No | 367/1431 (25.7%) | reference | | reference | |
|  | Yes | 25/87 (28.7%) | 1.21 (0.84-1.74) | 0.30 |  |  |
| Low birthweight | No | 373/1425 (26.2%) | reference | | reference | |
|  | Yes | 19/93 (20.4%) | 0.82 (0.50-1.33) | 0.41 |  |  |
| Maternal IPTp | SP | 186/781 (23.8%) | reference | | reference | |
|  | DP | 206/737 (28.0%) | 1.13 (0.91-1.41) | 0.27 |  |  |
| Education Level | None or primary | 330/1278 (25.8%) | reference | | reference | |
|  | O level or higher | 62/240 (25.8%) | 1.05 (0.77-1.41) | 0.78 |  |  |
| Housing type | Traditional | 300/1231 (24.4%) | reference | | reference | |
|  | Modern | 92/287 (32.1%) | 1.29 (0.98-1.70) | 0.07 |  |  |
| Household wealth | Poorest | 165/578 (28.6%) | reference | | reference | |
|  | Mid | 125/501 (25.0%) | 0.90 (0.69-1.18) | 0.46 |  |  |
|  | Highest | 102/439 (23.2%) | 0.93 (0.71-1.21) | 0.58 |  |  |
| Distance from clinic^c^ | ≥5 km | 315/1196 (26.3%) |  |  | reference | |
|  | <5 km | 75/307 (24.4%) | 0.84 (0.63-1.11) | 0.21 |  |  |

^a^Adjusted prevalence rate ratio, with adjustment for age, season, and infant Hb genotype. ^b^N=650 with Hb genotype; ^c^n=670 with household distance measured
